# Voxels Optimization in 3D Laser Nanoprinting

**DOI:** 10.1038/s41598-020-67184-2

**Published:** 2020-06-26

**Authors:** Yahya Bougdid, Zouheir Sekkat

**Affiliations:** 1 Department of Chemistry, Faculty of Sciences, Mohammed V University in Rabat, Rabat, Morocco; 2 Optics and Photonics Center, Moroccan Foundation for Advanced Science & Innovation & Research, MAScIR, Rabat, Morocco; 3 Department of Applied Physics, Osaka University, 2-1 Yamadaoka, Suita, Osaka 565-0871 Japan

**Keywords:** Optics and photonics, Laser material processing

## Abstract

Voxels, the 3D equivalent of 2D pixels, are obtained by individual point exposures in 3D laser nanoprinting, and are the building blocks of laser printed 3D micro/nano-structures, and their optimization is important in determining the resolution of printed 3D objects. Here, we report what is believed the first detailed study of the voxel size dependence on the z-potion of the laser spot in 3D nano-printing. That is, we study the evolution and the low-limit size (diameter and length) of voxels fabricated in the vicinity of the substrate/resin interface. We use two-photon absorption in a photopolymerizable resin, and we vary the position of the laser’s focal spot, with respect to the cover glass/resin interface; *i.e*. in the longitudinal direction (z-direction). We found that the minimum lateral and the longitudinal sizes of complete voxels depend on the extent of penetration of the laser focal spot inside the resin. Truncated voxels, which are fabricated by partial overlap of the resin and the laser spot, allow for the fabrication of nano-features that are not diffraction limited, and we achieved near 100 nm feature sizes in our 3D fabricated objects. Our work is of central interest to 3D nanoprinting, since it addresses the spatial resolution of 3D printing technology, and might have potential impact for industry.

## Introduction

The pioneering work of Kawata *et al*., about two decades ago, on 3-dimentional (3D) micro- nanofabrication by two-photon absorption (TPA) induced solidification of photoresists; i.e. two-photon polymerization (TPP), and the extensive works of Sun *et al*. led to a tremendous amount of research in the field owing to the potential of such a technology in enabling manufacturing at extremely small scales; *e.g*. nanoscales, with enhanced speed and flexibility^1,2^. Indeed, this technology, referred to now as 3D laser nanoprinting, matured into a widespread commercially available, reliable and versatile technology^3^. Perhaps the most important application of 3D nanoprinting is the realization of complex 3D structures with combinations of different materials to realize minute objects with multiple functionalities^4^, and mesoscale printing is also of importance^5^; and applications of printed 3D micro- nano-objects range from scaffolds for cell culture and tissue engineering^6–9^, photonic wire bonds^10^ and photonics crystals^11–13^, micro-optics^14^ and micro-mirrors^15^ and micro-lens systems^16,17^, metamaterials^18–21^, micro-machines^22–28^, and so on^29–33^. Important reviews summarize the field^34–44^.

While much of the efforts focused on developing efficient TPP materials^36,45^ and bringing the technology to a widespread use^3,4^, the wall dressed by the fundamental limits of the technology two decades ago still holds. A key feature of 3D nanoprinting is its intrinsic sub-diffraction limited (SDL) machining resolution capability^2^. TPP is based on TPA; *i.e*. a nonlinear optical (NLO) interaction of a laser light with a photosensitive medium (for example, photoresist) which is transparent at the exposure wavelength (for example, near-infrared, NIR). Due to the NLO effect of TPA, when a pulsed, for example femtosecond (Fs) laser, beam is tightly focused into the resin through an objective lens (OL) of high numerical aperture (NA), TPA photophysical and photochemical events can be localized within the focal spot volume; *i.e*. voxel. At the focal point, TPA results from the squared light intensity (SLI) distribution, which is spatially and temporally narrower than that of linear absorption. The tail of the laser focus induces a negligible solidification of the resin by TPA, and reduces the interaction volume of light with matter, thereby improving TPP resolution. Material’s nonlinearity, the so-called thresholding effect^2,40^, can further reduce the size of printed voxels, and finer features much smaller than the cube (*λ*^3^) of the laser wavelength can be fabricated. Using the threshold effect; e.g. using lower photon fluxes, leads to a reduced degree of crosslinking of the photoresist and to diminished mechanical stability of the voxels^46^. Compared to UV lithography, TPP resolution is better owing to a narrow spatial absorption and to the threshold effect; even-though this effect is partially compensated by the longer wavelength of the NIR laser commonly used in TPP.

Stimulated-emission depletion (STED) techniques can be used to further reduce the resolution of TPP beyond the diffraction limit of light. These techniques do not add extra nonlinearity, they deactivate a part of the actinic laser spot by using either adapted photochemistry by two-color single photon spatially controlled laser light^47^, or forced fluorescence in the photo-initiator instead of decay into radicals for TPP^39,48^; however their use in printing complex 3D objects has not been demonstrated. Technical tricks, exploiting the threshold effect, can also reduce the resolution of 3D laser nanoprinting. For example, the voxel size depends on the laser exposure dose, and increasing the scanning speed reduces the exposure time, and improves resolution, as it was demonstrated in, for example, a recent work where a ~*λ*/21; *i.e*. ~37 *nm*, resolution was achieved in TPP using a $780\,nm$ laser^49^. With such a resolution, only fibers could be achieved, and stable 3D structures were obtained with a ~*λ*/8.3; *i.e*. ~94 *nm*, resolution. The size of voxels is of much interest in 3D printing since it affects the resolution and sizes of printed objects, and the voxel’s connection to the glass substrate is important for the stability of the 3D objects through their adherence to the substrate during the development phase^50–52^. In this paper, we will discuss another important technique to further control the size of printed voxels. That is, the dependence of the voxel size on the z-position of the focus; a technique which is of central importance to TPP nanofabrication, especially given the long and intensive literature in this field. The study gives detailed information and quantitative analysis of the effect of the z-position of the laser focus spot (LFS) on the size of the fabricated voxels as well as the resolution of 3D nanoprinting.

## Materials and Methods

**Figure 1:**
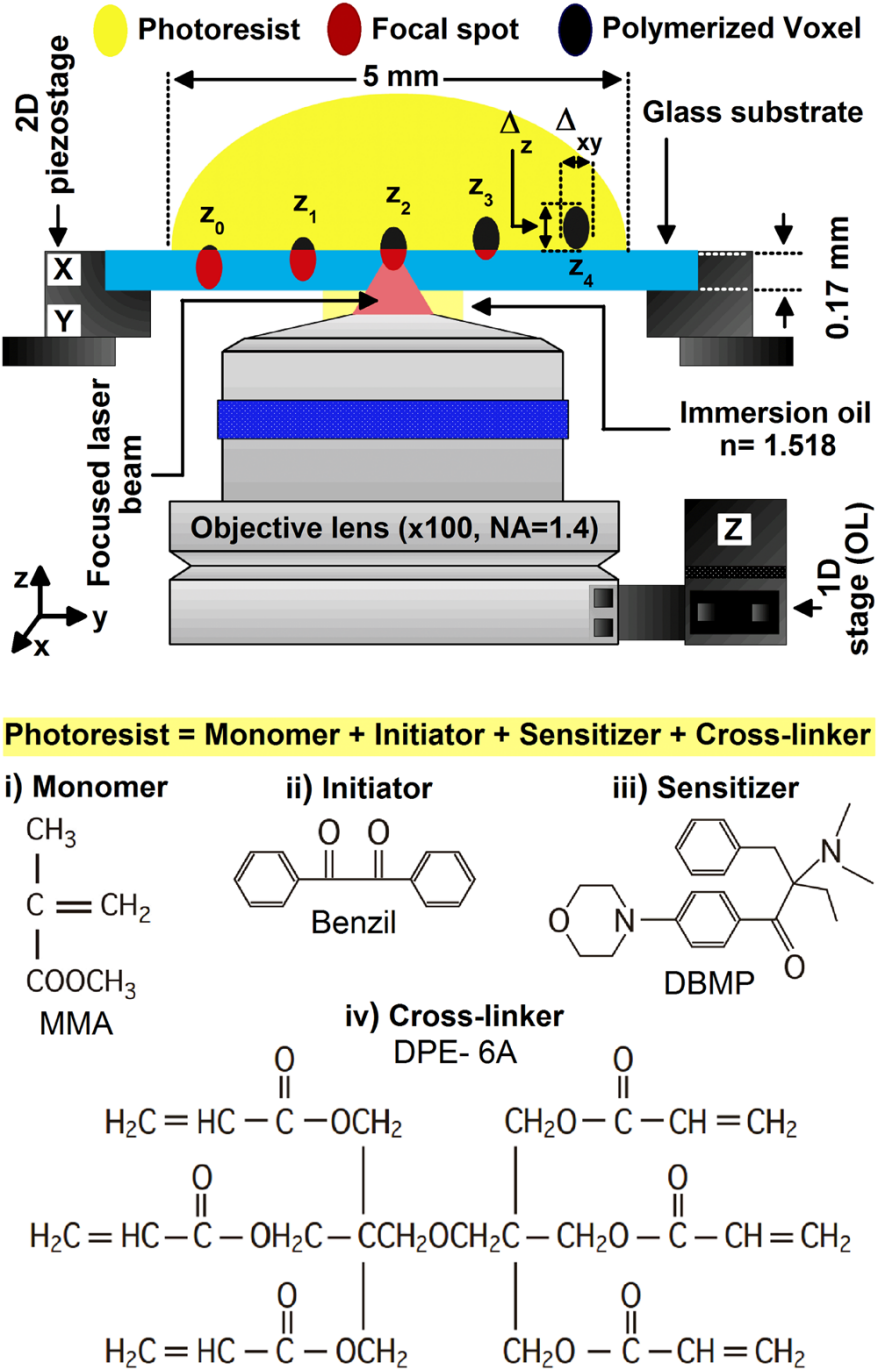
(Top) Schematic of the resin exposure configuration in our 3D laser nanoprinting system, indicating light focusing trough the objective lens and the variation of z-position of the laser focus in z-scan process: ${z}_{0}$, ${z}_{1}$, ${z}_{2},\,{z}_{3},\,{z}_{4}$. The resin is dropped on a glass slide attached to an x, y piezo-stage, and the lens is attached to a z-piezo scanner. The lateral ${\Delta }_{xy}$ and longitudinal ${\Delta }_{z}$ dimensions of the voxel are indicated, and the resin as well as the laser focus spot and the polymerized voxel are indicated by different colors. (Bottom) Structure formulae of the organic molecules which makeup the resin.

We used a negative acrylate resin for recording the laser focus in 3D. The resin was prepared by mixing the following chemicals. Methyl methacrylate (Monomer, Wako) (MMA) $(49\,wt\, \% )$, acts as the main skeleton of the nanofabricated structures; *i.e*. poly-(methyl methacrylate) (PMMA). DPE-6A (Cross-linker, Kyoeisya Chemical CO., LTD) $(49\,wt\, \% )$, promotes cross-linking and the hardness of structures. Benzil (photo-initiator, Wako) $(1\,wt\, \% )$ produces active species upon light excitation, and 2-Benzyl-2-(dimethylamino)-4′-morpholino-butyrophenone $97 \%$, (photo-sensitizer, Aldrich) $(1\,wt\, \% )$, is capable of absorbing light and transferring the excitation energy to the initiator. A viscous solution of the resin is prepared by mixing the above chemicals by steering overnight with a magnetic stirrer. The chemical structures of these compounds are shown in Fig. 1. Benzil is known as a good photo-initiator for photopolymerization of MMA^53,54^. It shows good absorption and photo-reactivity in the UV range, with high quantum yields for the generation of radicals, it is nontoxic and stable in the dark, and it is highly soluble in the resin. Resins based on acrylates are most commonly used for 3D printing owing to their ability of undergoing efficient photopolymerization upon TPA^34,50^.

The absorption spectrum (not shown) of the resin was measured by using a UV-Vis spectrophotometer (Perkin Elmer-Lambda 1050). The resin shows a strong optical absorption in the ultra-violet (UV) range, which is due to the $\pi$-$\pi$ bond of the benzil initiator, as well as the absorption by the sensitizer; and no absorption can be observed in the visible and NIR regions; *i.e*. almost $\sim 100 \%$ of transmittance. Therefore, to efficiently induce TPP, we used a Fs laser light with an irradiation wavelength of $\lambda =780\,nm$. Hence, the rate of TPA is proportionally related to the SLI; *i.e*. ${I}^{2}$, where $I$ symbolizes the light intensity of irradiation, and $2$ denotes the number of photons that are simultaneously absorbed during the TPP process. For the experiments discussed in this paper, we set the laser intensity above or equal to the TPP threshold intensity $({I}_{th} \sim 820\,kW.c{m}^{-2})$; e.g. the intensity below which TPP fabrication in the resin used is inefficient; e.g. resin not sufficiently cross-linked, and the fabricated structures are washed away during the development phase.

For 3D nanoprinting, we developed a custom-made optical system, which is described in detailed in^49^. Briefly, we employed, as laser source, a Ti: sapphire Fs laser system (Mai Tai, spectra physics) operating in mode-lock at $80\,MHz$ and $780\,nm$ with $140$-$fs$ pulses. The laser light was horizontally; e.g. Transverse magnetic (TM); i.e. x-polarized, before, and has polarization components along the y- and z- axes after, the OL^55–57^; and the actual resin is polarization insensitive unlike polarization sensitive materials^58,59^. Light polarization must play an important role in TPA when using polarization sensitive resins^60,61^. To induce TPP, the Fs laser beam was tightly focused by using an OL of high NA $(1.4)$ onto the resin that was dropped on a cover glass. An average power of $3\,mW$, corresponds to ${I}_{th}\, \sim \,8,2\,mW/\mu {m}^{2}$, for a beam diameter of 680 nm, corresponding to the beam waist at the laser wavelength *(vide infra)*, and to an energy per pulse of $37.5\,pJ$, and to a peak power per pulse of $\sim 268\,W$. The energy at the waist of the LFS can be estimated by considering the transmission of OL / cover glass system^44^. The TPP of the resin from liquid to solid results in a contrast of the resin’s refractive index; a feature which allows for monitoring *in situ* the fabrication by a charge-coupled device (CCD) camera. Refractive index of microstructures created by TPP can be measured with the interferometric technique using a Michelson interferometer^62^. During TPP printing of voxels, the resin, which is dropped on a cover glass, is positioned on an x,y piezo-stage, and the position of the laser focus was controlled along the z-direction of the OL, which is attached to a piezo-scanner. We use cover glass substrates because our optical set up is based on an inverted microscope. Both the sample stage and the OL were controlled by using a custom-made software. After TPP printing, the sample was rinsed in ethanol to remove the non-polymerized viscous resin, and then the remained polymerized structures were imaged with a field-emission scanning electron microscope (SEM) (FEI FEG 450). Several samples were prepared; e.g. voxels fabricated by TPP and developed and imaged with SEM.

## Results

**Figure 2:**
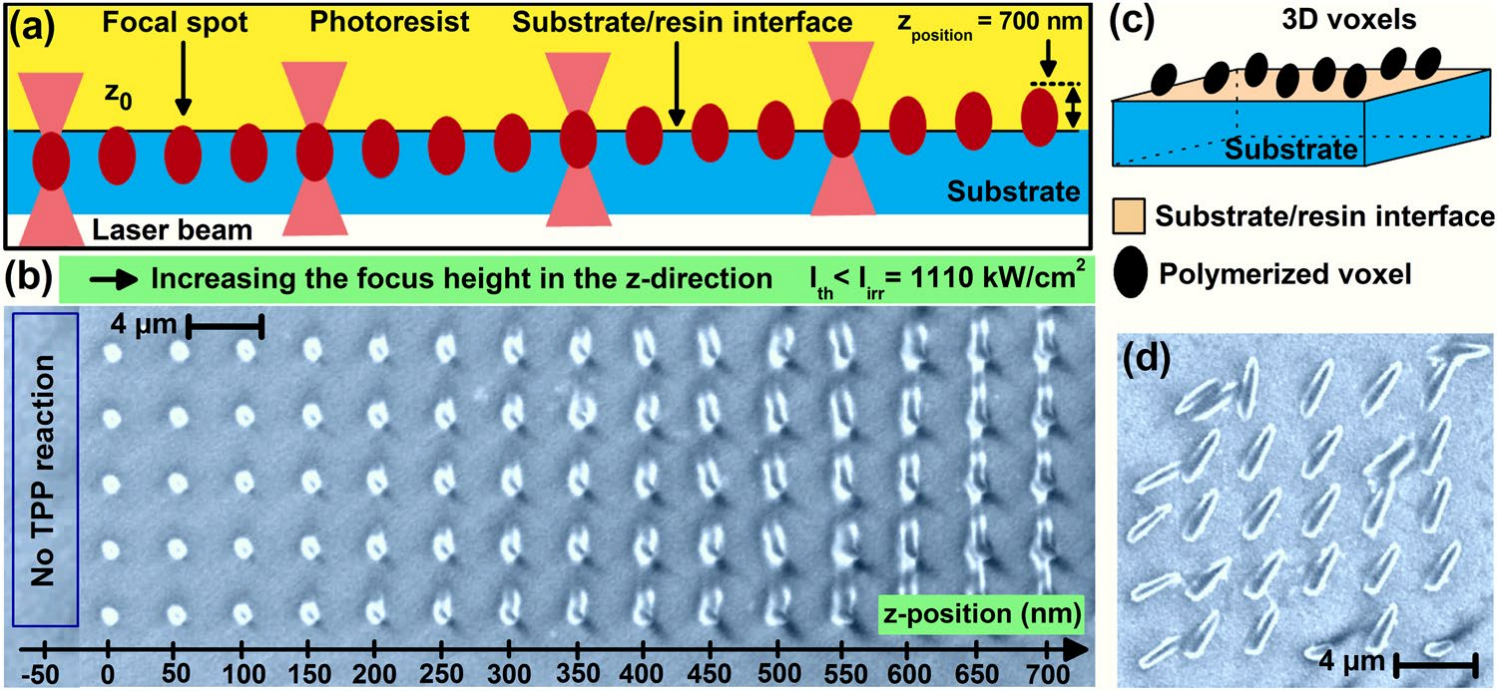
**(a)** Schematic illustration of the variation of the focused spot position along the z-direction during the z-scan process. The resin and the substrate and the focus spot are indicated. **(b)** Top-view SEM image of TPA polymerized voxels as represented in (**a**). **(c)** Schematic illustration of complete TPP voxels on the top surface of the glass substrate. **(d)** SEM image of printed voxels, schematically illustrated in (**c**), obtained for a z-position of the focus positioned at $\sim 1.4\,\mu m$ away from the interface. The typical laser intensity used in (**b,d**) was $\sim 1110\,kW/c{m}^{2}$.

To study the effect of the z-position of the LFS on TPP nanofabrication, we used an z-scan method whereby we first position the upper tip of the LFS near the glass/resin interface (position denoted by ${z}_{0}$), then we move it gradually into the resin as schematically depicted in Fig. 2(a). The LFS, after passing through the OL, is elliptically shaped with a diameter ($2{w}_{0}\, \sim \,1.22\lambda /NA\, \sim \,680\,nm$), and a longitudinal dimension; e.g. the depth of focus, which is twice the Rayleigh length, $2{z}_{R}=1.013\,\mu m$; with (${z}_{R}=\pi {w}_{0}^{2}/\lambda =506.5\,nm$) imposed by the laser wavelength; *e.g*. $780\,nm$, and the objective lens used; *e.g*. $(NA=1.4)$. The LFS penetrates into the resin by steps of 50 nm and 100 nm with a precision of 20 nm. In fact the precision of our piezo-stage is 1 nm, and for the actual experiments, we set our zero; e.g. ${z}_{0}$ at the first observable TPP fabricated voxel whereby the top of the tip of the LFS inside the resin is about 20 nm away from the glass/resin interface.

We performed a first experiment to study the shape of the voxel as a function of the z-position. We fabricated individual voxels at different z-positions of LFS with a $1\,ms$ laser exposure time and an average intensity of e.g. $I\, \sim \,1.11\,MW.c{m}^{-2}$. The step of the z-position was varied by 50 nm. Figure 2(b) shows SEM images of the voxels fabricated for different z-positions of the LFS at 5 different in-plane locations of ${z}_{0}$ which are separated by $4\,\mu m$. The formation of isolated voxels; *i.e*. complete voxels, is observed for large z-positions; *i.e*. larger than $1\,\mu m$ where the LFS is nearly fully located inside the resin. When the LFS is not too far away; i.e. z larger than $1.5\,\mu m$, from the interface, the full voxels are physically attached to the cover glass, as schematically depicted on Fig. 2(c) and shown on Fig. 2(d), and they remain on the substrate, with different spatial arrangements, after the development phase. Figure 2(b) also shows that the longitudinal size of the fabricated voxel increases with the increasing z-position, and, in particular, truncated voxels are observed for small z-positions. No fabricated structures were observed when z is smaller than ${z}_{0}$. The truncated voxels stick to and erect from the top of the cover glass, revealing their lateral size information; *i.e*. ${\Delta }_{xy}$. By printing complete voxels (Fig. 2(d)), both lateral $({\Delta }_{xy})$ and longitudinal $({\Delta }_{z})$ sizes can be measured.

In a second experiment, we studied the evolution of both the lateral and longitudinal sizes of the fabricated voxels with the z-position of the LFS. For this experiment, we set the laser’s average intensity to $({I}_{th}\, \sim \,820\,kW.c{m}^{-2})$, and we used 1 ms exposure time for each point exposure event, and we varied z by steps of 50 nm followed by steps of 100 nm, starting from ${z}_{0}$ up to $z=1.4\,\mu m$. We chose 1 ms, as minimum exposure duration, at ${I}_{th}$ to keep the fabricated voxels to minimum sizes, for a better precision. Exposure at ${I}_{th}$ ensures full TPP polymerization. In fact large exposure times and intensities lead to larger TPP fabricated voxels and may damage fabricated structures^34–36,49^.

**Figure 3:**
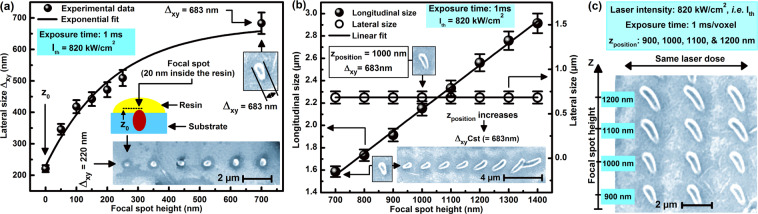
**(a)** Dependence of the ${\Delta }_{xy}$ on the z-position of the laser focus. Scatters are experimental data, and the solid line is exponential theoretical fit. The SEM micrograph of the corresponding voxels that are printed under different z-positions is shown as an inset. ${z}_{0}$ is indicated by an arrow on the data points and by a schematic in the inset **(b)** Longitudinal (black solid sphere) and lateral (blue open circle) size of voxels dependency on z-position of the focal spot. Scatters are experimental data and solid red lines are linear theoretical fits. **(c)** SEM image of voxels polymerized in different z-positions of the focal spot. Every three horizontal voxels were polymerized at the same focusing conditions; *e.g*. the laser dose and z-position of the focus. The exposure time and the laser intensity used for (**a–c**) were 1 ms and ${I}_{th}\, \sim \,820\,kW/c{m}^{2}$; respectively.

Figure 3(a) shows the evolution of the lateral size of the voxel, ${\Delta }_{xy}$, with the z-position of focus; and it follows an exponential law for z up to 700 nm owing to the SLI distribution, within the Rayleigh range, required for TPA. The closer the center of the LFS to the resin the stronger the TPP reaction. Figure 3(b) shows that ${\Delta }_{xy}$ saturates at; *i.e*.${\Delta }_{xy}\,=683\mathrm{nm}$ ._._ at z-position of $700\,nm$; a lateral size which is close to the theoretical diameter of the LFS; i.e. 680 nm *(vide infra)*. Indeed, at such a z-distance, more than 2/3^rd^, and consequently the diameter, of the LFS is fully located into the resin. Although we have no clear explanation as to why the experimentally observed lateral dimension of full voxels, obtained by near-threshold irradiation, are close to the theoretical size of LFS; it is very much plausible that it can be due to the nature of the photoresist used which favors efficient cross linking at low irradiation doses close to the threshold, given that ${\Delta }_{xy}$ and ${\Delta }_{z}$ depend on the irradiation dose and the type of photoresist used^63^. Indeed, we measured the evolution of ${\Delta }_{xy}$ with the energy dose by varying the laser intensity around ${I}_{th}$ for a fixed irradiation duration (1 ms) and z-position (420 nm), and we found that ${\Delta }_{xy}$ increases exponentially with the irradiation dose (figure not shown) in agreement with the literature^50,51,63^. For z-positions larger than 700 nm, ${\Delta }_{xy}$ remains unchanged, as intuitively expected for a given exposure dose, and the longitudinal size ${\Delta }_{z}$ changes appreciably; e.g. linearly up to $z=1.4\,\mu m$ (Fig. 3b). The same voxel size is obtained for the same z-position at different in-plane; e.g. lateral, positions (Fig. 3(c)). The longitudinal size of the voxel at $z=1.4\,\mu m$, is ${\Delta }_{z}\, \sim \,2.913\,\mu m$, and for z much larger than $1.4\,\mu m$, the voxels are washed away after the development phase. When the LFS is fully located inside the resin, the longitudinal size of the voxel exceeds $2{z}_{R}$; e.g. exceeds $1\,\mu m$, indicating that the resin in the close proximity of the LFS is also polymerized in the longitudinal; e.g. the propagation direction of the laser; a feature which could be explained by chain, or seed, photo-polymerization and/or self-guiding the laser light propagation caused by the solidified resin^64^.

Processing parameters play an important role in determining feature sizes of printed objects, and compromise of laser intensity and exposure duration as well as scanning speed dictates the resolution of TPP. For example, in our recent study, we showed that it is possible to fabricate nano-fibers that are ~37 nm thin by using high intensity and fast scanning speed, and the resolution we could achieve in stable 3D objects was ~ 94 nm^49^. The effect of the z-scan on the resolution of TPP nanofabrication can be seen at Fig. 3(a). Indeed, this figure shows that a lateral size of ${\Delta }_{xy}\, \sim \,220\,nm$ can be achieved at ${z}_{0}$ when only a small portion of the LFS reaches the resin with the exposure conditions discussed in the beginning of this section (see also the SEM image in the inset). The achieved ${\Delta }_{xy}\, \sim \,220\,nm$ feature size is better than predicted by Abbe’s diffraction limit, or lateral resolution, $(\lambda /2\,N.A.\, \sim \,279\,nm)$, and it is due to the fact that only the tip of the LFS reaches the resin (the diameter of the LFS at the glass/resin interface is smaller than $2{\omega }_{0}$) and to the optical nonlinearity nature of TPP; e.g. to the SLI law of TPA. Our study clearly shows that the minuteness of the feature size that can be fabricated by TPP depends on how small is the interaction volume of the LFS and the resin much like in STED techniques. 2D structures could benefit from nanofabrication with small z-positions to improve resolution; however, TPP is not required for 2D lithography and for 3D fabrication of architecturally simple structures, and high AR 2D structures can be produced by lithographic techniques with superior resolution such as e-beam lithography. Improving 3D-TPP resolution requires the development of TPP resins active in the ultra-violet (UV) range instead of the IR range; and the smaller the wavelength, the better the TPP resolution. Single photon polymerization is also an interesting alternative, especially with UV light since most materials exhibit appreciable absorption in the UV range.

**Figure 4:**
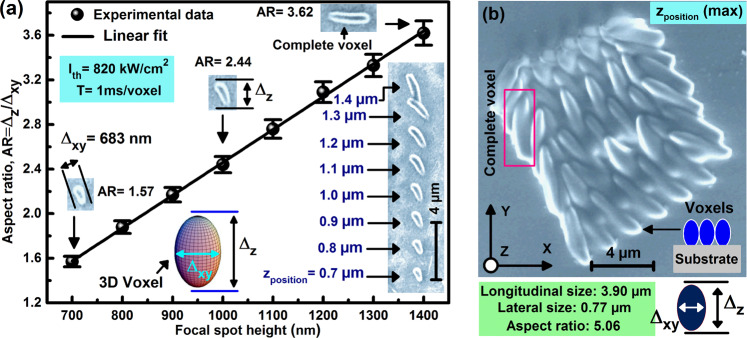
**(a)** Elongation factor; *i.e*. $AR={\Delta }_{z}/{\Delta }_{x,y}$. printed voxels versus the z-position of the laser focal spot. Scatters are the measured AR of nanoprinted voxels shown in the SEM image (inset), and the full line is a linear theoretical fits guide to the eye. **(b)** SEM micrograph of complete voxels that are polymerized far above the laser’s threshold intensity; *i.e*. at ${I}_{irr}\, \sim \,1650\,kW/c{m}^{2} > {I}_{th}\, \sim \,820\,kW/c{m}^{2}$.

The dependence of the AR; *i.e*. $AR={\Delta }_{z}/{\Delta }_{xy}$, of the fabricated voxels on the z-position of LFS is shown in Fig. 4(a). The experimental data points of this figure are adapted from ${\Delta }_{xy}$ and ${\Delta }_{z}$ in Fig. 3(b), and they show that the AR increases linearly with the increased z-position of LFS, ranging from 1.57, for a truncated voxel at $z=700\,nm$, to 3.62 for fully developed voxel at $z=1.4\,\mu m$. For our set up, Abbe’s axial resolution is $2\lambda /{(N.A.)}^{2}\, \sim \,796\,nm$; a resolution which is not as fine as the lateral one. For Gaussian beams, Abbe’s lateral resolutions may be corrected for the SLI associated with TPA, and a $\sqrt{2}$ factor should be introduced as $({d}_{xy}=\lambda /2\sqrt{2}\,N.A.\, \sim \,197\,nm)$), while the axial resolution ${d}_{z}$ is at least 2.5 times worse than ${d}_{xy}$ $({d}_{z}=2.5{d}_{xy}\, \sim \,394\,nm)$^39^. Voxels that are fabricated with large z-positions; *e.g*. z larger than $1.4\,\mu m$, are washed away during the development phase *(vide infra)*. When the laser intensity is increased above ${I}_{th}$, voxels are much larger; i.e. all ${\Delta }_{xy}$ and ${\Delta }_{z}$ and AR increase with the increased intensity, or irradiation dose. Indeed, Fig. 4(b) shows that voxels fabricated with a 1 ms exposure at $I\, \sim \,1650\,kW/c{m}^{2}$; e.g. nearly double of *I*_th_, exhibit ${\Delta }_{xy}=770\,nm$, and ${\Delta }_{z}=3.9\,\mu m$, and $AR=5.06$ larger than those observed with ${I}_{th}$ exposure for the same time and same z-position. We chose $z=1.4\,\mu m$ for Fig. 4(b) to compare with the largest voxel observed in Fig. 4(a). Clearly, ${\Delta }_{z}$ is more sensitive to exposure dose than ${\Delta }_{xy}$, and lower laser intensity at near-threshold exposure is advantageous to achieve voxels with lower AR and high spatial resolution.

**Figure 5:**
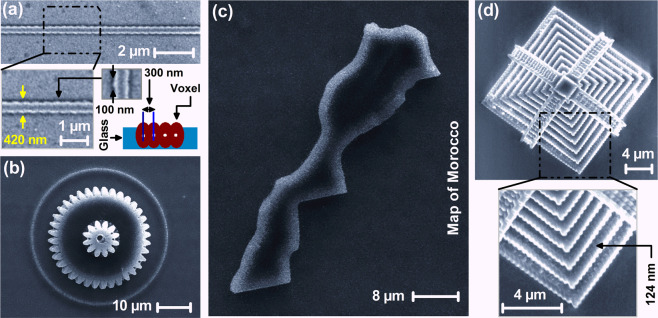
SEM images of 3D micros objects fabricated by using TPP-based 3D nanoprinting. **(a)** Nano-fiber of a 420 nm diameter; (inset) the overlapping of voxels; *i.e*. the scanning pitch in the x- and y- directions, was fixed to $\, \sim \,300\,nm$. **(b)** Top-view of a 3D micro-gear. **(c)** A microsized map of Morocco. **(d)** A 3D pyramid fabricated at sub-diffraction limit accuracy; *e.g*. ~124nm spatial resolution. The typical laser intensity and the focus z-position used in **(a–d)** are ${I}_{th}\, \sim \,820\,kW/c{m}^{2}$ and ${z}_{position}\, \sim \,120\,nm$ away from the interface; respectively.

To fabricate stable 3D objects with the finest possible resolution with our setup, we position the LFS at $z=100\,nm$ above ${z}_{0}$ (see Fig. 3(a)). At this position, we obtain a truncated voxel with a lateral size of ~ 400 nm, and a nano-fiber of 420 nm diameter with a ~ 100 nm feature size; i.e. the smallest fabricated feature (Fig. 5(a)). The exposure time was 1ms at ${I}_{th}$, and the lateral scanning pitch was 300 nm, so that voxels overlap laterally by 100 nm. The fiber is stable; i.e. remains on the substrate after the development phase. With this configuration, we also fabricated stable 3D nano-micro-objects by raster scanning layer by layer, with a pitch between layers in the z-direction of 500 nm, to insure a strong overlap between the voxels in the z-direction; e.g. overlap by the Rayleigh length of the LFS. The smallest feature size of our 3D objects varied between 100 and 124 nm (Fig. 5); a features size which is far below the TPA modified Abbe diffraction limit. A comment must be made about the resolution of TPP nanofabrication. Clearly, besides, the nonlinearity of TPA and materials thresholding and nanofabrication parameters, including the exposure dose and scanning speed, as previously reported in a long literature, our experimental results show that the resolution of 3D nanofabrication is strongly dependent on the spatial overlap of the LFS and the resin, and the scanning pitch for voxels connection which make up 3D structures. That is fabrication parameters play a major role in defining the resolution of 3D nanoprinting.

In summary, we investigated the influence of the laser-focus position along the z-direction on the resolution of 3D laser nano-printing by TPP. Using a systematic nanofabrication of voxels by varying the z-position of the LFS, we have experimentally examined their spatial dimensions; *e.g*. both lateral and longitudinal. We found that, while the voxel size depends on the laser’s exposure dose, its size is dictated by the extent of overlap between the LFS and the resin. By using truncated voxels having small aspect ratios, we could fabricate stable 3D micro-nano-objects, with feature sizes, near$\,100\,nm$, way below Abbe’s diffraction limit. TPP fabrication parameters play a major role in defining the resolution of 3D printed stable structures, and this study clearly demonstrates the impact of the z-position of the laser focus on the precision and the resolution of TPP nanofabrication; a feature which is of primary importance in 3D laser nanoprinting. Indeed, in most of the published literature, attention is not paid to the z-position and the overlap of the LFS and the resin, and our study, at the substrate / resin interface, demonstrates how important is the z-position in 3D nanoprinting. Perhaps, more studies should focus on this interface, by for example chemical treatment of the substrates for better adhesion of the resin, with the aim of using lower irradiation doses for better resolution. The resin it self is extremely important, from the points of view of materials nonlinearity and polarization sensitivity. Indeed, using polarization sensitive resins would allow for the fabrication of polarization sensitive structures for, for example, applications in diffractive, polarization sensitive, nano-optical devices.

## Data Availability

The authors declare that all data supporting the findings of this study are available from the corresponding author upon reasonable request.
